# Bone: A Neglected Endocrine Organ?

**DOI:** 10.3390/jcm13133889

**Published:** 2024-07-02

**Authors:** Anna Szeliga, Monika Grymowicz, Anna Kostrzak, Roman Smolarczyk, Gregory Bala, Katarzyna Smolarczyk, Blazej Meczekalski, Katarzyna Suchta

**Affiliations:** 1 Department of Gynecological Endocrinology, Poznan University of Medical Sciences, 60-535 Poznan, Polandankos30@op.pl (A.K.); 2 Department of Gynecological Endocrinology, Warsaw Medical University, 00-315 Warsaw, Poland; monika.grymowicz@wp.pl (M.G.); rsmolarczyk@poczta.onet.pl (R.S.); suchta.katarzyna@gmail.com (K.S.); 3 UCD School of Medicine, University College Dublin, D04 V1W8 Dublin, Ireland; greg.bala1@gmail.com; 4 Department of Dermatology Medical, University of Warsaw, 00-927 Warsaw, Poland

**Keywords:** bone, neuropeptide Y, parathormone, osteocalcin

## Abstract

Bone has traditionally been viewed in the context of its structural contribution to the human body. Foremost providing necessary support for mobility, its roles in supporting calcium homeostasis and blood cell production are often afterthoughts. Recent research has further shed light on the ever-multifaceted role of bone and its importance not only for structure, but also as a complex endocrine organ producing hormones responsible for the autoregulation of bone metabolism. Osteocalcin is one of the most important substances produced in bone tissue. Osteocalcin in circulation increases insulin secretion and sensitivity, lowers blood glucose, and decreases visceral adipose tissue. In males, it has also been shown to enhance testosterone production by the testes. Neuropeptide Y is produced by various cell types including osteocytes and osteoblasts, and there is evidence suggesting that peripheral NPY is important for regulation of bone formation. Hormonal disorders are often associated with abnormal levels of bone turnover markers. These include commonly used bone formation markers (bone alkaline phosphatase, osteocalcin, and procollagen I N-propeptide) and commonly used resorption markers (serum C-telopeptides of type I collagen, urinary N-telopeptides of type I collagen, and tartrate-resistant acid phosphatase type 5b). Bone, however, is not exclusively comprised of osseous tissue. Bone marrow adipose tissue, an endocrine organ often compared to visceral adipose tissue, is found between trabecula in the bone cortex. It secretes a diverse range of hormones, lipid species, cytokines, and other factors to exert diverse local and systemic effects.

## 1. Bone as an Endocrine Organ

On the 8 July 2022, Spanish scientists discovered the oldest human bones found to date on the European continent. Estimated to be around 1.4 million years old, the fragments provide enough anatomical resolution to be ascribed to the genus Homo. In humans, the skeleton is one of the largest organs of our body and contributes up to 15% of total body weight [1]. Of the many functions born by the skeleton, it is most often associated with those of protecting internal organs, functional and locomotor support, hematopoiesis, and calcium homeostasis. The extent of bone function and biology remains unclear, as recent discoveries suggest that bone plays a significant role as regulator of certain metabolic processes [2,3].

In humans, the newborn possesses 270 bones, which increases to around 355 during adolescence, eventually settling at approximately 206 bones in mature adulthood. Bone, as a highly dynamic organ within the human body, undergoes a continuous process of bone formation and resorption [4]. The average total weight of bone is approximately 10 kg in women and 12 kg in men. Comprising the skeleton are primarily three types of bone cells: osteoblasts, osteoclasts, and osteocytes, with osteocytes accounting for 95% of bone composition.

Postmenopausal osteoporosis was defined for the first time by Fuller Albright in 1941. He not only recognized the structural role of bone but also postulated on its metabolic activity. Bone undergoes constant remodeling, a process characterized by the interplay of osteoblasts and osteoclasts, with osteoblasts responsible for bone formation and the development of bone mass and strength. Osteoclasts, on the other hand, secrete protons and enzymes responsible for bone resorption.

Recent studies are beginning to uncover new and unexpected functions of the human skeleton [5]. Research in the 1970s demonstrated that bone produces at least five hormones: fibroblast growth factor 23 (FGF-23), lipocalin (LCN2), sclerostin, and osteocalcin, with the latter being understood to be the most powerful as a regulatory hormone. Osteocalcin (OCN), with a molecular weight of 6 kDa, has been a focal point of numerous studies over the years. Additionally, osteocalcin produced by osteoblasts plays a role in muscle function and strengthening through the release of IL-6 [6].

Nearly 20 years ago, Gerard Karsenty described the many roles of osteocalcin affecting organs such as the brain, pancreas, testes, and muscles. In particular, he described how it induces testosterone production by Leydig cells in the testes. Other studies have demonstrated its influence on glucose metabolism, insulin secretion, and insulin resistance [7]. Despite the majority of osteocalcin being located within the bone matrix [8], it accumulates in regions of the brainstem, thalamus, and hypothalamus, potentially binding with neurons. Furthermore, osteocalcin I is implicated in the synthesis of neurotransmitters such as serotonin, GABA, and dopamine. Studies have demonstrated the significant role it plays in motivation, mood regulation, and memory. Fibroblast growth factor 23 (FGF23) is a hormone-like protein secreted by osteoblasts and osteocytes. It functions both locally to regulate extracellular matrix mineralization and systemically as a participant in mineral metabolism. The FGF23 gene, spanning more than 8.5 kb, is located on chromosome 12 in humans [9]. Dipeptidyl peptidase 4 (DPP-4), also produced in osteoclasts, appears to be involved in functions related to glucose metabolism and the immune system [10]. Lipocalin-2 is implicated in the regulation of food intake and appetite within in the brain [11]. Sclerostin is produced mainly by osteocytes, and it acts as a paracrine regulator of WNT signaling and activity of osteoblasts and osteoclasts on bone surfaces. Moreover, sclerostin protein has been noted to act at a distance to regulate adipocytes, energy homeostasis, and mineral metabolism in the kidney [12,13,14].

As the study of bone formation and function continues to evolve, so will our understanding of bone function in the context of metabolic and endocrine regulation.

## 2. Materials and Methods

**Figure 1:**
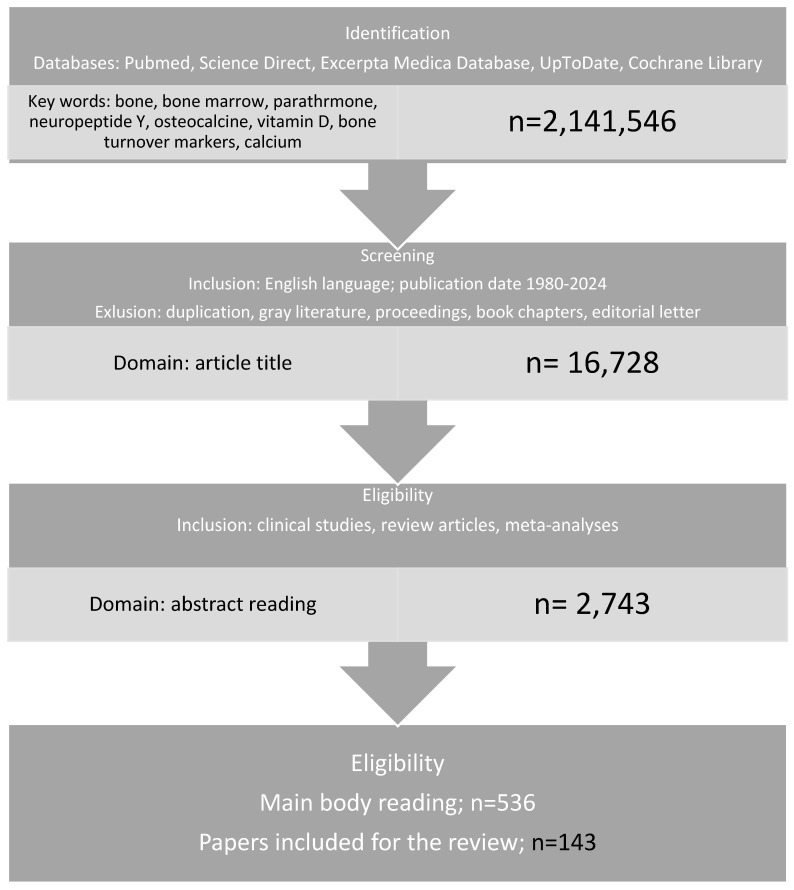
Materials and methods.

This review employed a comprehensive search across several major databases, including PubMed, ScienceDirect, Excerpta Medica Database, UpToDate, and the Cochrane Library. The search strategy utilized the following MeSH terms alone or in combination: bone, bone marrow, parathormone, neuropeptide y, osteocalcin, vitamin D, bone turnover markers, and calcium. The search was conducted between 1 January 2024 and 29 February 2024. All identified publications in English up to February 2024 underwent critical appraisal by the authors, with particular attention given to those directly relevant to the theme. Two of the authors independently read the titles and abstracts and classified them based on the section criteria. Duplications, proceedings, book chapters, and editorial letters were excluded. Then, the full texts from clinical studies, review articles, and meta-analyses published were systematically examined. Uncontrolled, nonrandomized, or case studies were excluded from the analysis. Additionally, reference lists of included articles were manually screened to further identify relevant studies (Figure 1).

## 3. Parathormone, Vitamin D, and Calcium—Mineral Metabolism

Bone metabolism includes processes such as bone formation, bone resorption, calcium homeostasis, and hormone regulation. Bones typically comprise 50–70% mineral and 20–40% organic material [15]. Calcium, phosphorous crystals, hydroxyapatite, and various ions such as sodium, fluoride, and magnesium constitute the mineral component.

Continuous osteogenesis and osteolysis facilitate bone remodeling and repair while assisting in maintaining calcium (Ca) and phosphorus (P). The skeletal system serves as an efficient reservoir for stored calcium and phosphate. Precise control of unbound extracellular ionic calcium (Ca^2+^) concentrations is essential for the normal functioning of cell membranes. Both calcium and phosphorus are independent and play essential roles in most disorders of bone mineral metabolism. Calcium has a fundamental role in muscle, blood, and nerve function, transferring information between cells and transmitting nerve impulses. Phosphorus is a constitute of biomembranes and nucleic acids, and many phosphorylated metabolites are necessary for diverse actions such as energy metabolism, cell differentiation, and cell proliferation.

**Figure 2:**
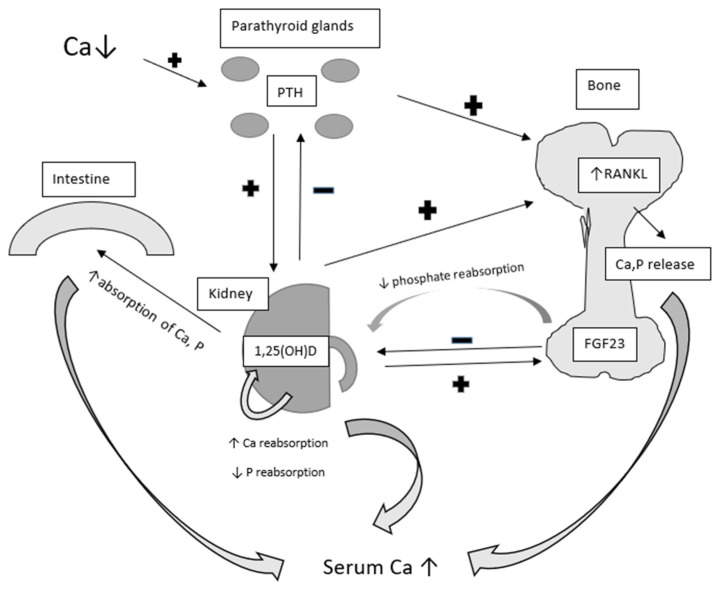
Interplay of PTH, vitamin D, and FGF23 in calcium and phosphorus level regulation. Ca—calcium, P—phosphorus, PTH—parathyroid hormone, 1,25(OH)D—1,25-dihydrocholecalciferol, RANKL—receptor activator for nuclear factor kB ligand, FGF23—fibroblast growth factor 23.

The principal molecules regulating blood calcium and phosphorus levels are parathyroid hormone (PTH) and vitamin D [16]. Other hormones influencing bone turnover include thyroid hormones, calcitonin, glucocorticoids, estrogens, and insulin-like growth factor 1 (IGF-1) [17]. When serum calcium levels fall below the normal range, the parathyroid gland increases the secretion of PTH. PTH will regulate calcium homeostasis through various mechanisms, primarily targeting bone and kidney function [18]. Specifically, parathyroid hormone acts on osteoblasts and osteocytes, upregulating the expression of RANKL (receptor activator for nuclear factor kB ligand). RANKL binds to its receptor, leading to maturation of osteoclast precursors and subsequent release of calcium into the bloodstream (Figure 2) [19].

Furthermore, PTH increases renal reabsorption of calcium and promotes the excretion of phosphate by regulating specific channels in the renal tubules [20,21]. PTH also upregulates the expression of 1-α-hydroxylase in the kidney, catalyzing the conversion of 25-hydroxycholecalciferol into its active form calcitriol (1,25-dihydroxycholecalcyferol) [22]. Vitamin D, functioning as a pro-hormone, undergoes complex endocrine regulation. It can be obtained from dietary sources or synthesized from cholesterol precursors in the skin layers under the influence of UV radiation. Subsequently, it is converted in the liver into 25-hydroxycholecalciferol (25(OH)D) and further metabolized in the proximal renal tubule into its active form 1,25-dihydroxycholecalciferol (1,25(OH)D).

1,25(OH)D directly acts on the small intestine to enhance the absorption of calcium and phosphate, stimulating bone resorption of these minerals. It also targets the kidneys to increase calcium and decrease phosphate reabsorption [23]. This opposite effect on phosphorus can be explained by tissue specific action of 1,25 (OH)D on sodium-phosphate cotransporter expression, enhancing it in the intestine and rather decreasing in the kidney. Fibroblast growth factor 23 (FGF23), primarily produced by osteocytes, plays a central role in regulating phosphorus homeostasis [24]. FGF23 acts as an endocrine regulator of renal phosphate transport by suppressing the expression of NaPi2a (sodium-dependent phosphate transport protein 2a; sodium-phosphate cotransporter 2a), thereby reducing phosphate reabsorption in the proximal collecting tubules. The drop in serum phosphate in turn depresses FGF23 expression and release from the bone.

The regulation of calcium and phosphate homeostasis involves an intricate array of interactions. While low calcium levels and increased PTH stimulate the production of 1,25(OH)D, elevated levels of 1,25(OH)D suppresses PTH secretion and promotes the conversion of vitamin D into its inactive form, 24,25-hydroxylated vitamin D (24,25(OH)D) [25], thus providing a protective mechanism against hypercalcemia. Vitamin D also enhances bone production of fibroblast growth factor 23, which in turn suppresses PTH expression in the parathyroid gland and reduces the production of active vitamin D while increasing the effect of 24,25(OH)D in the kidneys [26].

Although the primary role of vitamin D is in bone mineralization and calcium metabolism, its receptors modulate numerous target genes involved in various physiological and pathological processes, including cancer, immune modulation, cardiovascular diseases, and metabolic syndrome [27,28]. PTH has been shown to affect bioenergetic and metabolic processes in cells other than osteoblasts, including adipocytes and renal, skeletal, and cardiac muscle cells. This implicates PTH in numerous serious conditions beyond the skeletal system [20,29,30,31].

## 4. Osteocalcin and Its Profound Role

Osteocalcin, a γ-carboxyglutamic acid protein predominantly expressed and secreted by osteoblasts, plays a key role in bone endocrinology [32]. While osteoblasts are the primary source, small quantities of osteocalcin are also produced by odontoblasts of the teeth and hypertrophic chondrocytes [33]. Following transcription, osteocalcin undergoes posttranslational modifications within the osteoblast, including proteolytic cleavage of a prepropeptide and carboxylation of three glutamic residues (at positions 17, 21, and 24) into glutamic acid [34]. This carboxylation confers a high affinity for Ca^2+^ ions [35].

In bones, carboxylated osteocalcin is involved in bone mineralization and has been reported to inhibit hydroxyapatite growth during mineralization [35]. Additionally, it modulates the maturation of mineral species during osteogenic differentiation of mesenchymal stromal cells [36]. Osteocalcin functions as a negative regulator of bone formation, reducing osteoblast function and inhibiting osteoclastogenesis [37]. Nevertheless, a recent study found no significant differences in the cortical thickness between wild-type and osteocalcin-deficient mice [38].

Osteocalcin not only serves as a late protein product of osteogenesis affecting bone formation, but also acts as a signaling molecule to modulate the expression of transcription factors involved in osteogenic differentiation [36]. It also functions as a chemoattractant for osteoclast precursors [39]. The osteocalcin gene contains a “vitamin D-responsive element”, with vitamin D directly stimulating osteocalcin transcription, while vitamin K regulates the carboxylation processes [40].

Various cytokines, hormones, or growth factors can modulate osteocalcin production by modulating signaling pathways or interacting with transcription factors acting on the osteocalcin gene promoter region (BGLAP gene on chromosome 1q25–q31) [41]. Osteocalcin has been reported to function as a hormone regulating glucose metabolism, testosterone synthesis, muscle mass, brain development and function, and parasympathetic tone [35].

Osteocalcin plays a crucial role in glucose metabolism, as evidenced by studies in osteocalcin-deficient mice that exhibit increased insulin resistant and glucose intolerance compared to wild-type mice [37]. These deficient mice display elevated blood glucose levels and lower serum insulin levels. Furthermore, insulin secretion, sensitivity, and glucose tolerance are all diminished in osteocalcin-deficient mice, along with decreased energy expenditure [42]. This phenomenon may be attributed to decreased *β*-cell proliferation, reduced energy expenditure, and increased fat mass [43].

In adipose tissue, osteocalcin increases the expression of the ADIPOQ gene that encodes adiponectin, thereby enhancing insulin sensitivity [42,44]. This represents an indirect mechanism through which osteocalcin modulates bone function, as adiponectin stimulates the proliferation, differentiation, and mineralization of osteoblasts via the AdipoR1 and AMP kinase signaling pathways in autocrine and/or paracrine manners [43,45]. During bone resorption, osteoclasts release undercarboxylated osteocalcin into the blood circulation. Circulating osteocalcin, particularly its undercarboxylated fraction released during active bone resorption, directly impacts β-cells by stimulating insulin production [40].

### 4.1. Osteocalcin and the Gonads

Osteocalcin enhances testosterone production in Leydig cells by upregulating expression of genes encoding the enzymes necessary for its biosynthesis. It has been demonstrated that female mice deficient in osteocalcin maintain fertility and do not exhibit any gonadal abnormality [40]. Additionally, osteocalcin does not increase the expression of the genes necessary for the conversion of testosterone to estrogen [46]. Conversely, male mice with the same deficiency exhibit reduced volume in the testes, epididymis, and seminal vesicles, resulting in diminished reproductive activity [47]. These alterations are further associated with a 50% decrease in sperm count [40].

### 4.2. Osteocalcin and Cognition

Uncarboxylated osteocalcin plays a significant role in cognition by preventing neuronal apoptosis in the hippocampus [33]. It crosses the blood–brain barrier and binds to neurons in various regions, including the dorsal raphe in the brainstem, the ventral tegmental area in the midbrain, and the CA3 region of the hippocampus. Osteocalcin also serves as a stimulant for the synthesis of all monoamine neurotransmitters while reducing the synthesis of GABA, an inhibitory neurotransmitter [33]. Furthermore, osteocalcin signaling in neurons regulates the program of gene expression responsible for synthesizing these neurotransmitters [33].

## 5. Neuropeptide Y and Its Role in the Regulation of Bone Formation and Bone Resorption

Neuropeptide Y (NPY) is a 36 amino acid peptide discovered in 1983 [48]. It is primarily produced and expressed in the central nervous system, with the hypothalamus exhibiting the highest expression levels [49]. Peripherally, it is secreted by postganglionic sympathetic nerves [49]. NPY is also expressed in various peripheral tissue such as the pancreas, liver, heart, spleen, endothelial cells, and sympathetic nervous system [34].

NPY exerts a broad spectrum of actions, functioning as an orexigenic peptide, thereby increasing food intake, and promoting weight gain. Recent studies have identified NPY in bone tissue, particularly in osteoblasts, osteocytes, and adipocytes [50]. NPY is produced by osteocytes and osteoblasts [51], and its action at the bone level operates through autocrine and paracrine mechanisms. NPY and its receptors are also expressed in many tissues, playing typical regulatory roles in metabolic processes [52].

### 5.1. Neuropeptide Y and Bone Formation

NPY strongly impacts bone formation. Zhang et al. [53] found that NPY, through its receptor Y1R, can inhibit osteogenesis by suppressing Runx2 expression. Germline deletion of Y1R and knockout of NPY have been found to result in an anabolic effect on bone [54]. This effect is attributed to the upregulation of Runx2 and osterix levels, leading to a generalized increase in bone mass due to enhanced osteoblast activity and bone formation rate. Furthermore, NPY, through its anxiolytic effect, may offer protection against stress-induced bone loss, ultimately resulting in increased bone mass and formation rate. NPY can act indirectly by enhancing gap junction intercellular communication, thereby stimulating osteoblast osteogenic activity [55].

### 5.2. NPY and Bone Resorption

NPY has also been shown to influence bone resorption [56]. The process of bone resorption is primarily mediated by osteoclasts, which derive from hematopoietic stem cells. Wu et al. [57] observed that NPY stimulates osteoclast migration through Y1R and ERK1/2 activation. NPY suppresses isoprenaline-induced osteoclastogenesis by inhibiting RANKL expression in mouse bone marrow cells [58]. Park et al. [59] reported that NPY may be responsible for mobilizing hematopoietic stem/progenitor cells (HSPCs) from the bone marrow to the peripheral blood, thereby improving low bone mass in ovariectomy-induced osteoporotic mice by decrease osteoclast numbers.

### 5.3. Other Mechanisms of NPY Action on Bones

In addition to the aforementioned mechanism of NPY action on bone formation and resorption, NPY can exert other effects as well. NPY receptors (Y1R, Y2R, and Y5R) are expressed on endothelial cells [60], allowing NPY to indirectly influence bone. It has also been demonstrated that NPY upregulates VEGF, which in turn stimulates angiogenesis and osteoblastic differentiation [58]. NPY’s role in bone can be viewed from the perspective of the hypothalamus, its primary site of secretion. Chen et al. [61] conducted an insightful experiment investigating NPY as a mediator of the brain–gut–bone axis. They confirmed NPY’s participation in the axis, suggesting its potential as a novel target for postmenopausal osteoporosis treatments.

Matic et al. [51] generated mice with NPY overexpression specifically in mature osteoblasts and osteocytes. Their results suggest that osteoblast/osteocyte-derived NPY can modulate osteogenesis both in vivo and in vitro, significantly impacting bone formation. Analysis of studies on the relationships between NPY and bone reveals its potential role in the pathogenesis of osteoporosis, although it may have both a positive and a negative effect in this process. Long et al. [62] reported that NPY, through Y1R, may be involved in bone fracture healing. Furthermore, it was found that NPY is produced by immune cells such as macrophages and B cells.

Further studies are warranted to better understand the role of NPY in the process of bone formation and resorption. Such investigations would advance our understanding of this area and potentially open an avenue for the development of new drugs targeting NPY and its receptors in the treatment of bone diseases.

## 6. Bone Turnover Markers

To maintain integrity of bone structure, the intricate cooperation of osteoblasts, osteoclasts, and osteocytes is regulated by various substances released in an auto-, para-, or endocrine manner. In recent decades, the development of serum and urine assays for biochemical markers of bone metabolism has emerged, reflecting the enzymatic activities of osteoclasts and osteoblasts or breakdown products of bone tissue, which are valuable for both research and clinical practice [63].

Established biochemical markers of bone metabolism reflecting bone formation include serum total osteocalcin, bone-specific alkaline phosphatase (bone ALP), and the procollagen type 1 N-terminal propeptide (P1NP). Assessment of bone resorption include: pyridinoline (PYD), deoxypyridinoline (DPD), and fragments of the degradation of type I collagen (N- and C- telopeptides of type I collagen, or NTX and CTX). Furthermore, enzymes such as cathepsin or tartrate-resistant acid phosphatase type 5b (TRACP5b) reflect osteoclast activation (Table 1) [64].

Current bone turnover markers have some limitations, lacking tissue specificity and primarily reflecting the function of osteoblasts and osteoclasts rather than the activity of osteocytes. Osteocytes constitute most bone cells (90–95%) and play a principal regulatory role in all stages of bone metabolism [65]. Like endocrine cells, osteocytes possess the ability to detect variations in strain level and distribute signals leading to adaptive responses, thereby modulating the function of osteoclasts and osteoblasts at a molecular level [66,67].

The RANKL/RANK/OPG system is one of the main regulators of osteoclast formation and function. Osteoblasts and osteocytes produce RANKL, promoting the activation of the osteoclasts and bone resorption. Simultaneously, osteoblasts and osteocytes produce osteoprotegerin (OPG), a decoy receptor that blocks RANKL regulating the extent of osteoclast activation [68]. Osteoclasts can regulate osteoblasts by releasing proteins (e.g., transforming growth factor beta) or soluble factors (phingosine-1-phosphatase), or by direct contact through membrane-bound molecules [69]. Sphingolisine-1-phosphatase (S1P) promotes osteoclast differentiation by increasing RANKL production by osteoblasts and can serve as a biological maker of fracture risk [70].

The Wnt signaling pathway plays a central role in bone development, homeostasis, repair, and regeneration following injury [71]. Wnt produced by osteoclasts binding to its receptor (Frizzled) and co-receptors such as low-density-lipoprotein-receptor-related proteins (LRP5 and LRP6) activates their signaling pathway, leading to the accumulation of β-catenin in the nucleus and promoting the expression of numerous genes [72]. It has been demonstrated that Wnt binding to LPR5 and LPR6 can be inhibited by Dickkopfs (Dkk) proteins or sclerostin produced by osteocytes [73]. Serum DKK-1 levels are increased in osteoporosis, arthritis, bone metastases, and multiple myeloma [74]. Sclerotin, a highly specific protein of osteocytes coded by the SOST gene, reflects local bone production and is considered a major mediator for integrating mechanical, local, and hormonal signals sensed by the osteocyte [75]. By antagonizing the Wnt pathway in osteoblasts, sclerostin reduces bone formation. A genetically determined disorder of sclerostin synthesis in osteocytes may result in excessive bone formation [76], leading to conditions such as sclerosteosis or to van Buchen disease [77,78]. Sclerostin-deficient mice exhibit dramatically increased bone mineral density caused by intense bone formation and decreased osteoclast activity, while overexpression of sclerostin decreases bone mass and strength due to lower bone formation activity [79].

Fibroblast growth factor 23 is another potential bone turnover marker mainly produced by osteocytes. FGF 23 negatively regulates serum levels of inorganic phosphorous and 1,25(OH)D. Elevated levels of FGF23 have been observed in several skeletal disorders with mineralization abnormalities [80].

Monoclonal antibodies targeting the complex crosstalk of osteoblasts and osteoclasts have been developed. Denosumab, a monoclonal antibody against RANKL, is used as antiresorptive therapy, while romosozumab, a monoclonal antibody against sclerostin exhibits anabolic and antiresorptive properties [81]. Periostin (POSTN), mainly expressed by periosteal osteoblasts and osteocytes, is another candidate marker associated with the effects of mechanical factors and parathyroid hormone by modulating the Wnt signaling pathway and sclerotin expression [82]. Periostin may also be associated with the bone metastatic potential of lung and breast cancers [83].

MicroRNAs are composed of small noncoding RNAs and may play a significant role in the maturation and function of osteoblasts and osteoclasts. They play an essential role in regulating gene expression and exert their action extracellularly through blood circulation or secreted microvesicles [84]. Numerous studies have reported on the influence of microRNAs on every stage of osteogenesis, regulating the interplay between different cell types in bone. However, our understanding of how microRNAs regulate bone homeostasis is yet to be fully established [85,86,87].

## 7. Bone Marrow Adipose Tissue (MAT) and Mesenchymal Stem Cells (MSCs) as Endocrine and Immunomodulatory Systems

**Figure 3:**
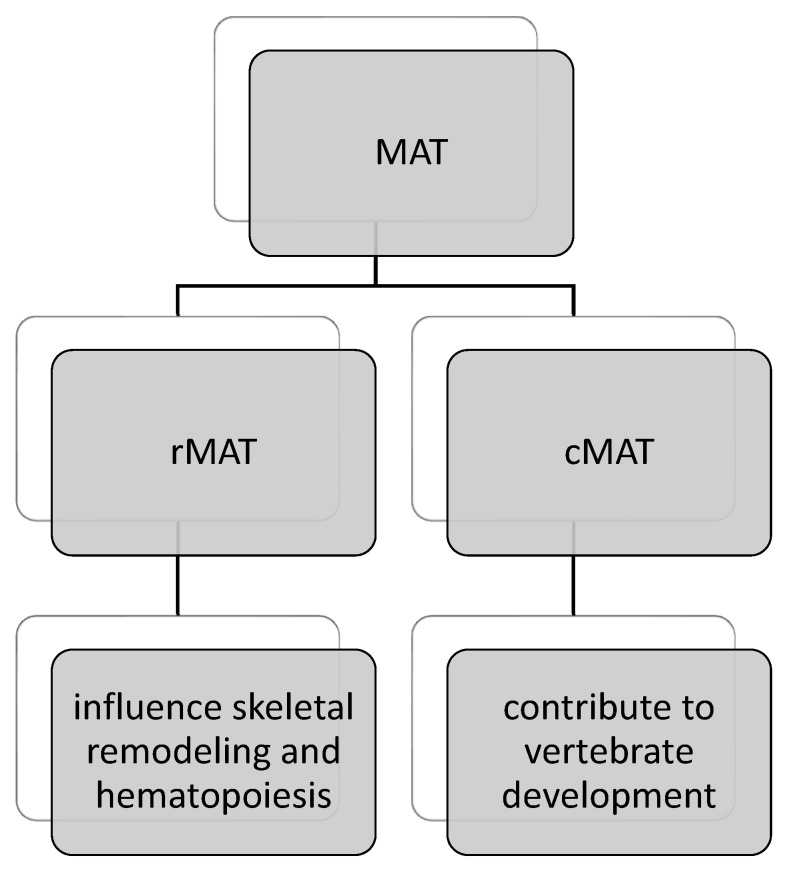
Division of bone MAT (marrow adipose tissue). rMAT—regulated MAT; cMAT—constitutive MAT.

Marrow adipose tissue (MAT) is located within the marrow cavity of bones, constituting approximately 70% of bone marrow and accounting for 5–10% of the total fat mass in lean, healthy adults [88,89]. The distribution of MAT is concentrated in peripheral bones of the arms and legs, with lower concentrations in centrally located bones [90]. Two distinct types of MAT can be identified: regulated MAT (rMAT) and constitutive MAT (cMAT), each characterized by a different lipid profile and gene expression pattern. While cMAT may contribute to vertebrate development, rMAT is believed to influence skeletal remodeling and hematopoiesis (Figure 3) [91].

Despite MAT being recognized for over a century, its role remains incompletely understood. Recent research has emphasized its endocrine function, both locally and systemically. Increased MAT levels have been associated with various health conditions, including type 1 diabetes, osteoporosis, aging, Cushing syndrome, anorexia nervosa, estrogen deficiency, and caloric restriction, as well as radiotherapy and chemotherapy for cancer [92,93,94,95]. Similarly, certain pharmacological agents such as glucocorticoids, thiazolidinediones, and fibroblast growth factor-21 have been linked to MAT accumulation [96,97,98].

Conversely, decreased MAT levels may be observed in conditions such as hypertensive heart failure, lipodystrophy, or Gaucher’s disease, as well as in states of chronic caloric restriction. This observation suggests that MAT accumulation may initially occur as an adaptation to starvation, serving as a fuel source during chronic and terminal phases [99,100,101,102].

**Figure 4:**
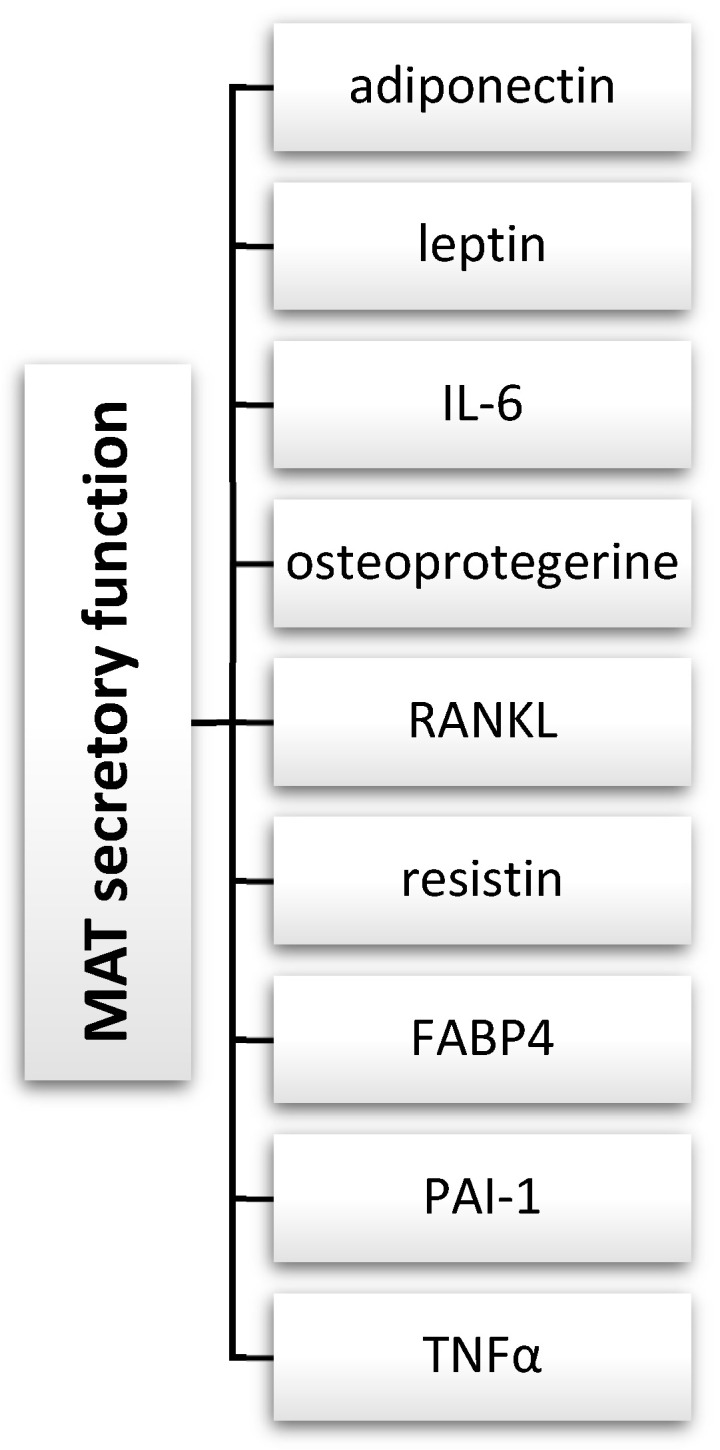
MAT secretory function. IL-6—interleukin 6; RANKL—receptor activator for nuclear factor κ B ligand; FABP4—fatty acid-binding protein 4; PAI-1—human plasminogen activator inhibitor-1; TNFα—tumor necrosis factor α.

Given its adaptive nature and the variability in secretory mediators over time, MAT may influence hormonal homeostasis locally and systemically. Numerous studies have explored the secretory function of MAT, as detailed by Sulston and Cawthorn [103]. The authors described numerous endocrine and paracrine factors expressed or secreted by bone marrow adipocytes, such as adiponectin, leptin, TNFα, PAI-1, FABP4, resistin, RANKL, osteoprotegerine, and IL-6 (Figure 4). An increase in adiponectin secretion by MAT may lead to elevation of its serum concentration in certain health conditions. Adiponectin may locally impact skeletal muscles, but it can also have systemic effects such as increasing glucose tolerance, improving hepatic insulin sensitivity, or enhancing cardiovascular function. Additionally, it may exhibit anti-tumor and anti-inflammatory effects.

**Figure 5:**
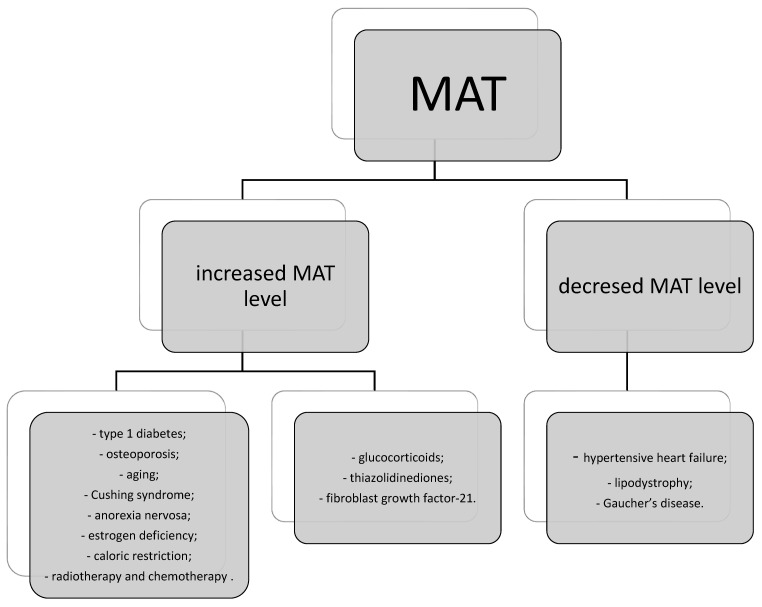
Factors and states that influence bone MAT level.

Both adiponectin and leptin are involved in skeletal remodeling, as receptors for both adipokines are present on osteoblasts, osteoclasts, and precursors of osteoclasts [104,105]. MAT activity increases adiponectin serum levels and promotes skeletal muscle adaptation, while the influence of caloric restriction on adiponectin levels remains unknown (Figure 5) [78].

Different subtypes of MAT also contain different lipid profiles. The level of fatty acid saturation varies depending on the health condition of the individual in question. Patsch JM et al. in their research showed that an increased level of fatty acid saturation in MAT is correlated and can be a predictive factor of bone fracture risk [106].

In their research, Sulston R. and Cawthorn W. [103] also indicate numerous endocrine and paracrine functions of MAT. According to the authors, apart from systemic endocrine effects, MAT may also impact bone loss by activating mechanisms responsible for decreased formation and/or increased resorption. MAT’s secretory function may also suppress hematopoiesis and enhanced tumor growth, survival, or invasiveness in certain conditions, for example, by lipid transfer to tumor cells.

Bone marrow mesenchymal stem/stromal cells (MSCs) are non-hematopoietic multipotent stromal cells that can differentiate into osteoblasts, chondrocytes, and adipocytes (marrow adipose tissue). Marrow adipocytes are derived from MSCs [107]. On the other hand, adipocytes are recognized to inhibit osteoblast activity and contribute to osteoclastogenesis [106,108,109,110]. Therefore, targeting MSC pathways towards osteogenesis instead of adipogenesis seems to have potential for skeletal regeneration [111].

Bone marrow mesenchymal stromal cells produce many immunomodulatory molecules such as nitric oxide, PGE2, indoleamine 2,3 dioxygenase, or IL-6 and have the ability to inhibit proliferation and activity of macrophages, neutrophils, NK cells, mast cells, and dendritic cells. Therefore, it has been postulated that MSCs may be able to reduce inflammation and be helpful in autoimmune diseases or transplant rejection treatment [112,113,114,115,116,117,118,119].

The bone marrow is regarded as a fundamental component of long-lasting immunological memory, providing protective immunity after infection or vaccination and also taking part in chronic inflammation. The bone marrow is isolated and privileged compartment for long survival of dormant cells responsible for our immunity. Long-lived memory plasma cells were identified in the bone marrow [120].

There is accumulating evidence that also resting and dormant populations of memory lymphocytes T and B are maintained in bone marrow niches [121,122,123].

Maintenance and control of the number of plasma cells and lymphocytes in the bone marrow clearly depends on cellular contact with individual mesenchymal stromal cells, presenting high heterogeneity and expressing different subsets of cytokines and chemokines [124]. Recruitment of memory plasma cells of the bone marrow indicate long-lasting humoral protection, while memory lymphocytes T and B mobilized into blood in secondary immune reactions indicate the strength of reactive memory [125]. Research on bone marrow immunological memory niches may in future allow the development of new effective vaccines and novel therapies against immune-mediated diseases.

As the authors indicate, the road ahead is long if we are to fully understand the role of MAT and MSCs in the human organism and to utilize such knowledge for therapies. Most research regarding MAT and MSCs is based on cell cultures, in vitro studies, or animal models. Despite advances in the field, many questions remain unresolved.

## 8. Novel Bone-Derived Substances

### 8.1. Osteopontin

Osteopontin (OPN) is a highly phosphorylated extracellular matrix glycoprotein with several identified binding sites for arginine-glycine-aspartic acid, heparin, integrin, thrombin, calcium, and metalloproteases [126]. Osteopontin is released by osteoclastic and osteoblastic cells and plays a pivotal role in the binding of the osteoclasts onto the bone surface, modulating their function [127]. It is postulated that OPN reduces the risk of bone fractures by suppression of over-mineralization of the tissue [128]. Osteopontin was also detected in many other tissues and organs and is considered to have other functions such as modulation of inflammatory response and wound healing processes, increase in cell migration in cancer, prevention of nephrolithiasis, and increase in insulin resistance and artheroclerosis [129,130,131].

### 8.2. Lipocalin 2

Lipocalin 2 (LCN2) is another glycoprotein upregulated in response to bacterial infection and tissue injury [132]. It has been demonstrated that osteoblasts are the primary source of LCN2 [133]. By activating anorexigenic pathways in the hypothalamus, LCN2 suppresses appetite [11]. High levels of lipocalin 2 are associated with obesity, dyslipidemia, and insulin resistance and can be considered as a biomarker for metabolic disorders [134].

### 8.3. Other Novel Bone-Derived Factors

There are other bone- derived factors, such as N-linked glycoproteins (SIBLINGs), including osteopontin (OPN), dentin matrix protein-1 (DMP1), bone sialoprotein (BSP), matrix extracellular phosphoglycoprotein (MEPE), and dentin sialophosphoprotein (DSPP). Nevertheless, further studies need to be carried out to assess the influence of these substances in human homeostasis [34].

## 9. Impact of Bone Hormones on Other Tissues and Organs

Bone is specific organ that plays very important role in human physiology. Bone, for a long time, was viewed as a static organ that does not present interactions with other organs and tissues. At present, this view has been changed, and bone is presented as a very active organ that has an important impact on other tissues and organs like muscles, the brain, the immune system, blood vessels, the pancreas, the kidneys, and the liver [135].

An important interaction is between bone and muscles. Osteocalcin has influence on insulin sensitivity at the muscle level. Additionally, osteocalcin causes adaptation to exercise by stimulation of IL-6 production [136]. TGF-beta (which is mainly bone origin) can have an impact on muscle activity regulation [137].

Influence of bone on the brain was previously mentioned. For instance osteocalcin can reach different brain structures (hippocampus, substantia nigra) and regulate neurotransmitter secretion [138]. Lipocalin can cross the blood–brain barrier and influence the anorexigenic pathway (through the melanocortin 4 receptor) [133].

Important crosstalk refers the influence of hormonal factors from bone on the immune system. RANKL can be regarded as a very important factor that links both systems. It is responsible for the immunological response and development of immune organs (secondary lymphoid organs) [68]. The role of osteoblasts in this aspect is also important. They participate in the differentiation of T cells and B cells in the bone marrow [139].

There is also the impact of hormonal factors from bone on the pancreas. Uncarboxylated osteoglycin in serum presents a negative correlation with insulin resistance, obesity, and diabetes [140]. The link between bone resorption markers and diabetes also was observed. There are studies that showed that resorption markers were higher in diabetes mellitus patients than in non-diabetic subjects [141].

The impact of bone (through different hormones secreted by bone) on the kidneys should also be stressed. It was reported that increased levels of sclerostin and DKK1 can cause impairment of kidney function [142].

The liver is another organ that can be influenced by hormonal factors secreted by bone. Hepatic osteodystrophy is related to bone loss and associated with chronic liver disease. It was reported that serum sclerostin levels were lower in nonalcoholic steatohepatitis patients than in healthy donors [143].

The crosstalk between bone and different organs plays an interesting and unique role in the human body, which was studied and identified during recent years.

This provides great opportunity not only for recognizing new mechanisms of this crosstalk but also for the development of new diagnostic and therapeutic procedures of systemic diseases.

## 10. Conclusions

Bone is an organ that plays an important role in endocrine metabolism. Further research is necessary to determine the exact relationships between bones, secreted hormones, and other endocrine organs. Determining their exact effects may be of great importance in further diagnostic and treatment options.

## Figures and Tables

**Table 1:** Bone turnover markers.

Bone Formation Markers	Bone Resorption Markers
Serm total osteocalcin	Pyridinoline (PYD)
Bone-specific alkaline phosphatase (bone ALP)	Deoxypyridinoline (DPD)
N-terminal propeptide of type 1 collgen (P1NP)	C-telopeptide of type 1 collagen (CTX)
	N-telopeptide of type 1 collagen (NTX)
C-terminal propeptide of type 1 collagen (P1CP)	Tartrate-resistant acid phosphatase type 5b (TRACP5b)

## Abbreviation

1,25(OH)D	calcitriol (1,25-dihydroxycholecalcyferol)
25(OH)D	25-hydroxycholecalciferol
bone ALP	bone-specific alkaline phosphatase
BSP	bone sialoprotein
Ca	calcium
Ca^2+^	ionic calcium
CTX	C-telopeptides of type I collagen
Dkk	Dickkopfs proteins
DMP 1	dentin matrix phospoprotein 1
DPD	deoxypyridinoline
DPP-4	dipeptidyl peptidase 4
DSPP	dentin sialophosphoprotein
ERK	extracellular-signal-regulated kinase
FABP4	fatty-acid-binding protein 4
FGF-23	fibroblast growth factor 23
GABA	gamma-aminobutyric acid
HSPCs	hematopoietic stem/progenitor cells
IGF-1	insulin-like growth factor 1
IL-6	interleukin 6
LCN 2	lipocalin 2
LPR	low-density lipoprotein receptor
MAT	marrow adipose tissue (rMAT regulated MAT; cMAT constitutive MAT)
MEPE	matrix extracellular phosphoglycoprotein
MSCs	mesenchymal stromal/stem cells
NaPi2a	sodium-dependent phosphatase transport protein 2a; sodium-phosphate cotransporer 2a
NPY	neuropeptide Y
NTX	N-telopeptides of type I collagen
OCN	osteocalcin
OPG	osteoprotegrin
OPN	osteopontin
P	phosphorus
P1CP	C-terminal propeptide of type 1 collagen
P1NP	N-terminal propeptide of type 1 collagen
PAI-1	human plasminogen activator inhibitor-1
POSTN	periostin
PTH	parathyroid hormone
PYD	pyridinoline
RANK	receptor activator for nuclear factor kB
RANKL	receptor activator for nuclear factor kB ligand
S1P	sphingolisine-1-phosphatase
TNFα	tumor necrosis factor α
TRACP5b	tartrate-resistant acid phosphatase type 5b
VEGF	vascular endothelial growth factor
Y1R, Y2R, Y5R	NPY receptors
